# Tissue Structure as a Primary Factor Influencing Vascular Sealing: Results of an Ex Vivo Study on Porcine Carotid Arteries

**DOI:** 10.3390/bioengineering13070719

**Published:** 2026-06-24

**Authors:** Andreas Kirschbaum, Dimitri Raico, Florian Kirschbaum, Moritz Jesinghaus, Nikolas Mirow

**Affiliations:** 1 Department of Visceral, Thoracic, and Vascular Surgery, University Hospital Giessen and Marburg (UKGM), Marburg Campus, Baldingerstrasse 1, D-35043 Marburg, Germany; 2 General Surgery, Eichhof Hospital, Eichhofstrasse 1, D-36341 Lauterbach, Germany; raico.dmitri@gmail.com; 3 Department of Human Medicine, Albert Ludwig University of Freiburg, Breisacher Strasse 153, D-79110 Freiburg im Breisgau, Germany; florian.kirschbaum007@gmx.de; 4 Institute of Pathology, University Hospital Giessen and Marburg (UKGM), Marburg Campus, Baldingerstrasse 1, D-35043 Marburg, Germany; moritz.jesinghaus@uk-gm.de; 5 Department of Human Medicine, Philipps University of Marburg, Baldingerstrasse 1, D-35043 Marburg, Germany; nikolas.mirow@t-online.de

**Keywords:** bipolar vessel sealing, elastin content, burst pressure, porcine carotid artery, histological vessel structure, ex vivo study

## Abstract

Bipolar vessel sealing systems are widely used in surgery, yet their effectiveness varies depending on the histological composition of the target vessel. In particular, the influence of elastin on seal stability is not well understood. Porcine carotid arteries, which show a pronounced proximal–distal elastin gradient, provide an ideal model for systematic analysis. In this study, fresh porcine carotid arteries were divided into three segments based on vessel diameter (<5 mm, 5–7 mm, >7 mm). Histological EvG staining was used to quantify elastin and collagen content. All vessels (n = 8 per group) were sealed using a bipolar marSeal^®^ 5 plus device, followed by burst pressure testing and peel force measurements. Elastin content increased significantly from peripheral to central segments (9% → 25% → 42%; *p* < 0.001), while collagen content remained constant (22 ± 2%). In parallel, seal stability decreased markedly: burst pressures dropped from 723 mmHg to 240 mmHg and to 31.5 mmHg (*p* < 0.001). Peel forces showed the same trend (1.75 ± 0.07 N → 0.65 ± 0.03 N → 0.26 ± 0.11 N; *p* < 0.001). Wall thickness showed no proportional relationship to seal quality. Interestingly, the sealing performance of bipolar systems seems to be greatly influenced by the histological structure of the vessel wall. A high elastin content—rising from 9% to 42% along the carotid artery—was associated with a reduction in burst pressure and peel strength. These findings highlight the need to consider tissue composition when selecting sealing methods and support the development of adaptive energy delivery technologies.

## 1. Introduction

Reliable sealing of blood vessels is a key component of numerous surgical procedures [1,2] and is becoming increasingly important, particularly in invasive surgery [3,4,5,6,7]. Modern bipolar sealing systems enable fast, reproducible, and material-saving hemostasis and have established themselves in many areas of surgery as an alternative to conventional ligatures [8,9,10,11,12]. Despite their widespread use, however, the quality of the seal produced is not consistent, but is influenced by a variety of anatomical and biophysical factors [13]. While the influence of vessel diameters and wall thickness has already been described in several studies, the role of tissue structure, particularly the proportion of elastic fibers, in the stability of the seal has not yet been sufficiently clarified. Arteries show significant differences in their histological structure depending on their location and functional load [14,15,16]. Elastic arteries such as the aorta have a high proportion of elastin fibers, while muscular arteries in the periphery mainly contain smooth muscle cells [17,18,19,20]. The common carotid artery is a vessel with a pronounced proximal–distal elastin gradient [21] and is therefore particularly well suited for systematically investigating the influence of tissue structure on the quality of the seal. Previous experimental work [22,23,24] suggests that elastin-rich vessels exhibit lower burst pressures after thermal sealing. However, as yet there has been a lack of studies quantifying this relationship along a clearly defined anatomical vessel course while taking mechanical stress parameters such as peel forces into account. Against this background, the present ex vivo study investigates whether and to what extent the sealing quality changes along with the porcine carotid artery and whether these changes are primarily determined by the vessel dimension or the histological structure. For this purpose, three vessel segments with characteristically different diameters and elastin contents were defined, and both burst pressures after bipolar sealing and peel forces were measured in a standardized manner. In addition, a quantitative analysis of the collagen and elastin content was performed using histological EVG staining.

The results of this study should contribute to a better understanding of the biomechanical principles of bipolar vessel sealing and highlight the possible limitations of the technique in elastin-rich vessels. This is relevant not only for experimental research but also for clinical application, as insufficient sealing quality, especially in larger, elastic arteries, can increase the risk of intraoperative complications. By combining histological analysis and mechanical testing, the study provides new insights into which tissue parameters significantly determine the stability of a sealing suture.

## 2. Materials and Methods

The heart–lung package was removed from freshly slaughtered pigs (EU standard: 90 kg). The aortic arch with the branches of the two carotid arteries was located. The carotid arteries were severed centrally at the aortic arch and dissected free in their further course to the periphery. The perivascular sheath tissue was completely removed. The specimens were rinsed clean with NaCl solution and packed in moist compresses. They were immediately transported to our research laboratory in a cool box at +4 °C. The transport time to the laboratory was a maximum of 10 min. In the laboratory, the diameters of the carotid arteries were measured using a digital caliper.

The following three vessel sections were identified according to their outer diameter: Group 1, less than 5 mm (periphery), Group 2, 5–7 mm (middle carotid), and Group 3, greater than 7 mm (aortic carotid). In addition, the wall thickness of the individual vessel sections was determined in [mm].

**Figure 1:**
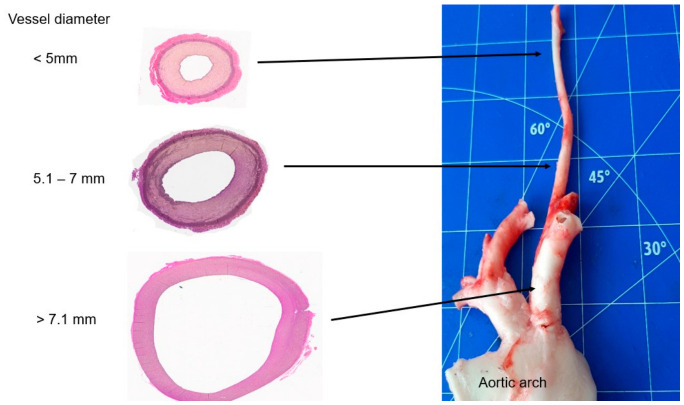
Course of the porcine carotid artery from the aortic arch to the periphery; histological cross-sections of the different groups of vessel diameters (Elastica Van Gieson staining, Institute of Pathology, University of Marburg).

The entire carotid artery was divided into three specimens, each 2.5 cm long, according to outer diameter. A vascular ring from each specimen was sent to the Institute of Pathology at the University of Marburg for histological examination. There, the vessels were stained with hematoxylin–eosin [HE] and Elastica–Van–Gieson [EVG]. Figure 1 shows the course of the porcine carotid artery from the aortic arch to the periphery with examples of histological vascular cross-sections (EVG staining in each case). The proportion of elastin and collagen in the vascular cross-section (in the EVG staining) was determined by image analysis of the characteristic colors using the GSA Image Analyzer Version 4.4.1 program (www.gsa-online.de). Eight histological stains were evaluated per group. Elastin content was quantified from fully digitized EvG-stained cross-sections (4x magnification). The entire vascular cross-section was defined as a region of interest; background and edge artifacts were masked. To identify elastin, which is characteristically black in EvG staining, a specific RGB color value range was defined based on representative fibers. Software then classified all pixels in the cross-section based on these color thresholds. The percentage of the area occupied by elastin was calculated as the ratio of elastin pixels to the total number of pixels. Finally, the segmentation was visually verified using a color-coded overlay to rule out misclassifications.

**Figure 2:**
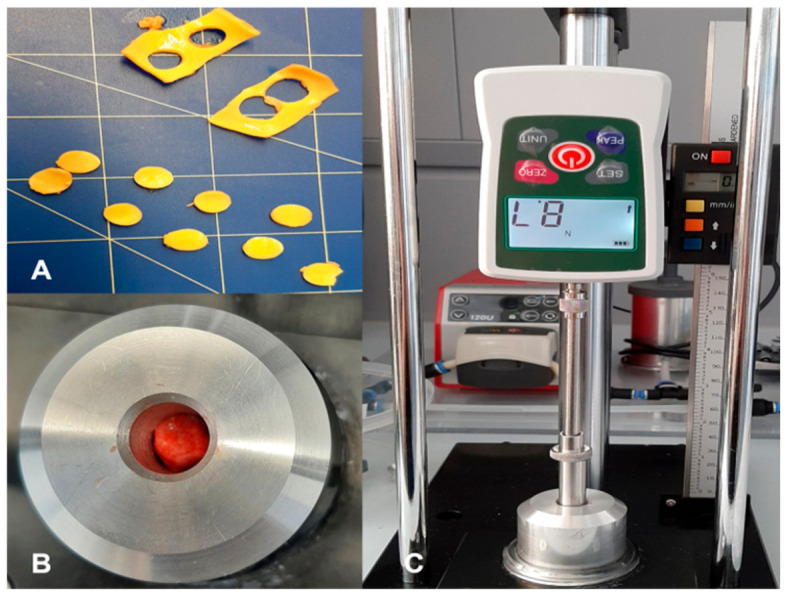
Performing axial compression on a vessel disk: (**A**) vessel disks punched out of the vessel wall; (**B**) vessel disk inserted into the compression channel; (**C**) setup for compressing the vessel disk with a digital force gauge in front [N] and right next to it a digital display showing the reduction in the height of the vessel disk [mm] (own photograph).

To measure the uniaxial controlled compression of the vessels, the preparations from each group were cut lengthwise and stretched out on a cork board with pins. A round disk was punched out of each vessel using a punch (diameter 10 mm). This disk was inserted into a metal cylinder with a 10 mm channel milled into it. A piston was inserted into the channel. The vessel disk was compressed step by step with increasing compressive forces. The maximum compressive force was 500 N. The decrease in the height of the vessel disk could be read on a digital display. The values of this uniaxial controlled compression were determined by eight vessel disks from each of the three groups. Figure 2 shows the experimental setup.

Vessels from each group (n = 8 each) were sealed using the bipolar sealing instrument marSeal^®^ 5 plus (KLS Martin SE & Co. KG, Tuttlingen, Germany), which was connected to a maXium^®^ sealer generator (KLS Martin SE & Co. KG, Tuttlingen, Germany). The vessels were severed mechanically using a blade integrated into the instrument. The burst pressures were then determined. To do this, a connector was inserted into the vessel lumen and secured with a cable tie. The connector was connected to a closed pump system which had been filled with water beforehand. The system was then calibrated, and the intravascular pressure was continuously recorded by a pressure gauge. After starting the pump, the intravascular pressure increased until the seal burst. This burst pressure was recorded and documented. All vessels in the three groups were examined. Mean values and standard deviations of the individual mean values were determined using an online data analysis and statistics program called numiqo (https://numiqo.com). For group comparisons, the mean values of the burst pressures were compared using a nonparametric test appropriate to the small number of cases. A significance level of *p* < 0.05 was assumed.

**Figure 3:**
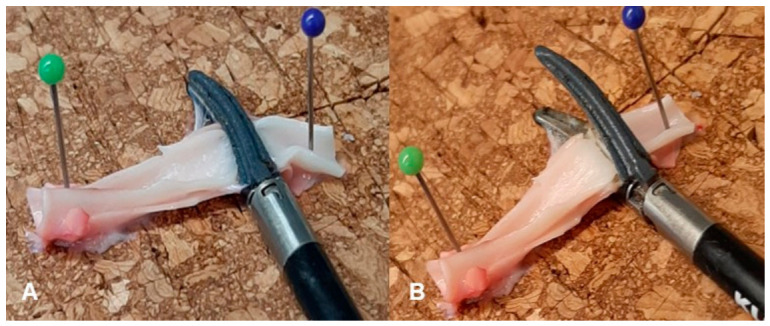
(**A**) Sealing of vessel strips fixed to a cork plate with pins using the bipolar sealing instrument marSeal^®^ 5 plus (KLS Martin SE & Co. KG). (**B**) Result after sealing the vessel stripes; the sealing instrument is open and shows the sealing zone (own photograph).

To examine whether only the tissue structure and not the vessel dimensions were relevant to the sealing quality, a peeling test was also performed. Again, eight vessels per group were used. The vessels, which were 2.5 cm long, were opened lengthwise. Strips with a width of 5 mm each were formed. Two strips from the same group were placed on top of each other and fixed to a cork plate at the edges with pins. They were then sealed in the middle using the bipolar sealing instrument marSeal^®^ 5 plus (KLS Martin SE & Co. KG, Tuttlingen, Germany), which was connected to the maXium^®^ sealer generator (KLS Martin SE & Co. KG) (see Figure 3).

**Figure 4:**
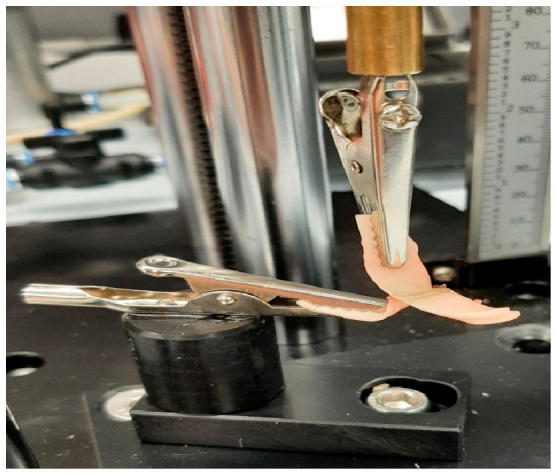
Principle of the peel test. The two sealed strips of tubing are pulled vertically upward by hand. The peel force is displayed on a digital force gauge as the strips of tubing are separated from each other (own photograph).

The sealed vessel strips were then clamped into a device with clamps, and the upper strip was pulled vertically upward manually. An integrated force gauge recorded the maximum force [N] when the two strips were separated. We refer to this force as the peel force. Please refer to Figure 4, which illustrates the peel test.

The mean values and standard deviations of the peel forces for each group were calculated using the above data analysis and statistics program numiqo. Differences between the groups were determined using the nonparametric Mann–Whitney U test. Significance was set at *p* < 0.05. Additionally, we used Kruskal–Wallis testing for the three groups and a post hoc-corrected pairwise test.

From each group, several seals were sent for histological examinations (HE and EVG staining).

## 3. Results

The vessels smaller than 5 mm (n = 8) had an outer diameter (OD) of 4.85 ± 0.01 mm. Wall thickness in this group was 0.5 ± 0.01 mm. For vessels between 5.1 and 7 mm, the mean outer diameter was 5.8 ± 0.05 mm. Wall thickness in this group was 0.6 ± 0.02 mm. The mean outer diameter of vessels larger than 7 mm was 10.7 ± 0.04 mm. Wall thickness in this group was measured at 0.75 ± 0.03 mm (see Table 1).

The average proportion of elastin, determined by the color analysis program on the Elastica–Van–Giemson sections, was 9 ± 0.5% for vessels smaller than 5 mm, 25 ± 1% for vessels between 5.1 and 7 mm, and 42 ± 2% for vessels larger than 7 mm. The groups differed significantly in this respect (*p* < 0.001). Collagen content was constant in all groups at 22 ± 2%.

**Figure 5:**
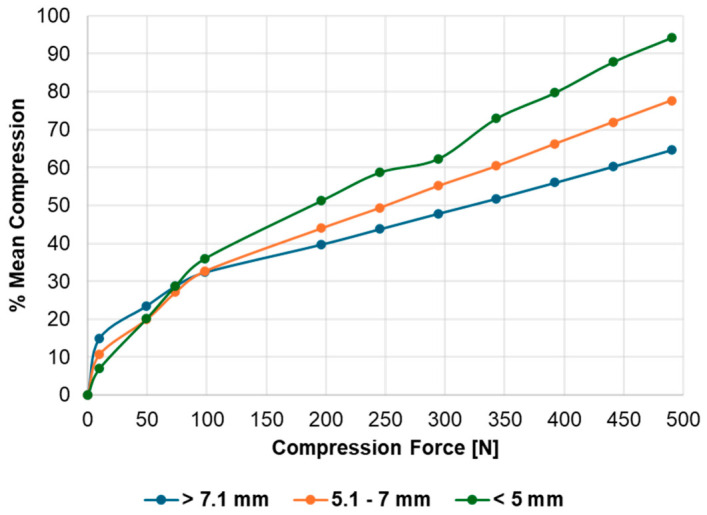
Graphical overview of the % average compression as a function of the compression force [N] of the individual groups 1–3 (n = 8/group in each case) (own results).

The mean % compression of the vessel disks in the individual groups showed clear differences. In the group 1 of carotid arteries < 5 mm, the mean % compression at 490 N was 94.25 ± 3.14%. For carotid arteries measuring 5.1–7 mm (group 2), the mean % compression at the same force was 77.68 ± 7.36%. The mean % compression for vessels > 7 mm (group 3) was only 64.66 ± 7.76%. Table 2 and Figure 5 show an overview of these results.

The difference in measured burst pressures when comparing the individual groups was also highly significant (*p* < 0.001). Mean burst pressures were 723 ± 37.5 mmHg for vessels smaller than 5 mm, 240 ± 18.75 mmHg for vessels between 5.1 and 7 mm, and 31.5 ± 3.75 mmHg for vessels larger than 7 mm. Kruskal–Wallis testing indicated a significant difference between the categories of the independent variable with respect to the dependent variable, *p* ≤ 0.001. Based on the available data, the null hypothesis was therefore rejected. Using a Dunn–Bonferroni test, groups were compared in pairs to determine significant differences. Comparison of groups one and three resulted in an adjusted *p*-value of less than 0.05. Therefore, based on the available data, the two groups differed significantly. Pearson correlation analysis showed a very strong negative correlation between the percentage of elastane and burst pressure. This correlation was statistically significant, r = −0.97, *p* < 0.001. Determination of peel forces showed similar trends. For vessels smaller than 5 mm (n = 8), the mean peel force was 1.75 ± 0.07 N. Vessels between 5 and 7 mm showed a mean peel force of 0.65 ± 0.08 N. For larger vessels over 7 mm, the mean peel force was only 0.26 ± 0.11 N. Comparison of the groups revealed highly significant (*p*< 0.001) differences (see Table 3).

Pearson correlation analysis showed a very strong negative correlation between the percentage of elastane and peel strength. This correlation was statistically significant, r = −0.95, *p* < 0.001.

**Figure 6:**
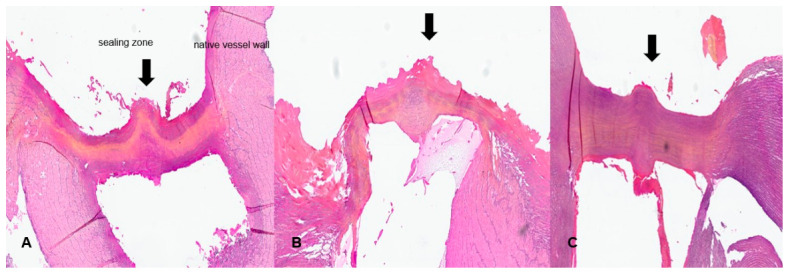
Comparison of sealing zones (marked with arrow) after EVG staining: (**A**) Group 1 (<5 mm), (**B**) Group 2 (5.1–7 mm), and (**C**) Group 3 (>7 mm) (Institute of Pathology, University of Marburg).

Histological evaluation of the sealing zones showed that the anatomical structures in the peripheria were intact. Centrally, there is a coagulated area in the contact zone of the two stripes. Figure 6 shows a comparison of the three sealing zones of two vessel strips in each group.

## 4. Discussion

This ex vivo study clearly indicates that the quality of bipolar vessel sealing depends on the histological structure of the vessel wall and is not primarily determined by geometric parameters such as diameter or wall thickness. Elastin content appeared to strongly influence the mechanical stability of the sealing seam. This finding is of considerable importance both for understanding the underlying biomechanical mechanisms and for the clinical application of thermal sealing systems. Bipolar sealing systems are based on thermally induced denaturation and the recombination of collagen fibers, which form a homogeneous and resilient fusion zone if exposed to heat. Elastin, on the other hand, exhibits significantly lower thermal reactivity and contributes little to the formation of a stable sealing seam. Previous experimental work on elastic arteries such as the aorta or pulmonary artery has already provided evidence that elastin-rich vessels exhibit lower burst pressures after thermal sealing [22,24]. However, there have been a lack of systematic studies along a defined anatomical vessel course that quantitatively assess the influence of the elastin content while additionally considering peel forces as mechanical stress parameters. While the present data demonstrate a strong association between increasing elastin content and decreasing seal stability, this relationship cannot be interpreted as exclusive or causal. Vessel diameter and, in particular, tissue compressibility—as shown by our disk-compression experiments—also contribute to sealing performance and may interact with elastin-related effects. As the study did not include size-matched muscular arteries with low elastin content, the influence of elastin cannot be isolated from other structural parameters. We therefore consider elastin a contributing rather than a singular determinant of sealing quality. Future studies will incorporate visceral arteries of comparable diameter to directly test for further insight into the role of elastin content in sealing performance.

Due to its pronounced proximal–distal elasticity gradient, the porcine carotid artery is an ideal model for investigating this relationship [21,25]. Our present data not only confirm previous observations, but also significantly expand upon them: for the first time, it has been shown that the elastin content along a single vessel is directly and almost linearly related to the quality of the seal. The combination of histological analysis, burst pressure measurement, and peel testing provides a robust and consistent evidence base. The significant increase in elastin content from peripheral to central (9%, 25%, and 42%) was associated with a drastic decrease in burst pressures and peel forces. These findings can be explained biomechanically. Elastin has high elasticity and resilience [25], which means that a larger proportion of the wall structure remains mechanically active even after thermal exposure. Unlike collagen, elastin denatures only to a limited extent, so it contributes little to the formation of a homogeneous fusion zone. With elastin, the resulting seal is mechanically less stable, and increased elastic tension leads to earlier mechanical insufficiency. These mechanisms explain low burst pressures in the proximal carotid segments, which only reached 31.5 ± 3.75 mmHg despite the greater wall thickness. In the context of physiological loading conditions, the burst-pressure values observed in this study must be interpreted against the normal arterial pressures of adult pigs. In healthy 90 kg swine, systolic arterial blood pressure typically ranges between 120 and 150 mmHg, with mean arterial pressures between 90 and 110 mmHg. If compared with these physiological values, the sealing performance of the different carotid segments demonstrates a structural dependency. Peripheral, muscular segments (<5 mm) reached burst pressures of 723 mmHg, exceeding physiological systolic pressures by a factor of five and therefore providing a substantial safety margin. Mid-carotid segments (5–7 mm) achieved 240 mmHg, still surpassing physiological systolic pressure but with a markedly reduced reserve. In contrast, proximal, elastin-rich segments (>7 mm) exhibited burst pressures of only 31.5 mmHg, far below the physiological range. These findings indicated that bipolar sealing was mechanically reliable only in muscular, low-elastin arteries, whereas central arteries were not safely sealed, as their burst-pressure thresholds were well below the in vivo hemodynamic load. The different stability between sealed proximal and distal parts of the vessels may be related to differences in elastin content. In our experimental setup, this seemed to make a relevant difference regarding sealing quality. On the other hand, wall thickness expressed no proportional correlation in this regard, which underscores the dominance of histological factors. The supplementary peel test is an important methodological component, as it largely eliminates geometric influencing factors and isolates tissue interaction for consideration [26,27,28]. Significantly lower peel forces in elastin-rich vessels (1.75 N, 0.65 N, 0.26 N) indicate that the adhesive strength of the seal is primarily determined by the tissue structure. The consistent results from burst pressure measurement and peel testing significantly strengthen the validity of the study and show that the elastin content is a consistent and reproducible predictor of seal quality. These results are of great clinical significance. Bipolar sealing systems are widely used in visceral, thoracic, and vascular surgery [10,29,30,31,32]. While they enable reliable hemostasis in smaller, muscular arteries, the available data show that their effectiveness is significantly limited in elastic arteries. This applies to central vessels such as the common carotid artery, the subclavian artery, or the aorta, which have a high elastin content.

These vessels carry an increased risk of intraoperative sealing insufficiency leading to postoperative bleeding. The results therefore clearly indicate that alternative procedures, such as ligatures, clips, or stapling systems, should be preferred for use on vessels rich in elastin. In addition, the data provides an important impetus for further development of bipolar sealing systems. Possible options include adaptive energy profiles that take tissue structure into account, sensor-based systems that measure elasticity or wall tension in real-time, modified pressure profiles to stabilize the fusion zone, or combined procedures that integrate thermal and mechanical components. A quantitative description of the relationship between elastin content and sealing quality provides a valuable basis for this. Despite its clear results, the study has some limitations. The ex vivo procedure employed allows for standardized examination but does not reflect all physiological conditions of the living organism. Factors such as blood flow, temperature or vascular tension may influence the sealing quality in vivo. Another limitation is that only porcine carotid arteries were examined, which, although similar to arteries of the human vascular system [28], are not identical in all possible aspects.

Furthermore, the number of cases per group is limited due to the experimental effort involved; nevertheless, it is statistically sufficient. Finally, only a single bipolar sealing system was examined, so the results primarily apply to this system.

## 5. Conclusions

This ex vivo study indicates that the sealing quality of bipolar vessel sealing systems is not mainly influenced by parameters such as vessel diameter and wall thickness. Instead, the histological structure of the vessel wall appears to be of the utmost importance. In particular, elastin content seems to be a limiting factor for the mechanical stability of vessel seams. Even though the results of this study are based on ex vivo experiments, they illustrate that, when using bipolar systems, elastin-rich vessels can presently only be sealed with limited reliability. To avoid bleeding risk, for these arteries, alternative techniques should be employed. Our results provide an important impetus for the further use and development of adaptive sealing technologies.

## Figures and Tables

**Table 1:** Comparison of the mean outer diameters and wall thicknesses of the individual groups (n = 8 specimens each) (own results).

Groups (n = 8 Specimen Each)	External Diameter (mm)	Wall Thickness (mm)
1 (<5 mm)	4.82 ± 0.01	0.5 ± 0.01
2 (5.1–7 mm)	5.8 ±0.05	0.6 ± 0.02
3 (>7.1 mm)	10.7 ± 0.04	0.75 ± 0.03

**Table 2:** Comparison of the mean % compression of groups 1–3 (n = 8 specimens each) as a function of the respective compression force (own results).

Compression Force [N]	0	9.5	49	73.5	98	196	245	294	343	392	441	490
Group 1 % mean compression SD±	0	6.99 ± 2.74	20.16 ± 2.99	28.83 ± 3.3	36.04 ± 3.35	51.31 ± 3.81	58.75 ± 3.37	62.83 ± 6.24	72.97 ± 3.84	79.75 ± 3.74	87.86 ± 4.33	94.25 ± 3.14
Group 2 % mean compression SD±	0	10.73 ± 4.74	19.95 ± 7.44	27.15 ± 7.68	32.67 ± 8.55	43.99 ± 7.18	49.39 ± 7.18	55.16 ± 7.53	60.4 ± 7.53	66.26 ± 7.92	72.01 ± 7.51	77.68 ± 7.36
Group 3 % mean compression SD±	0	15.09 ± 5.26	23.56 ± 4.99	28.78 ± 5.41	32.41 ± 5.68	39.76 ± 7.21	43.89 ± 7.51	47.94 ± 7.12	51.85 ± 7.45	56.04 ± 7.86	60.39 ± 8.27	64.66 ± 7.76

SD = standard deviation.

**Table 3:** Overview of the average peeling forces for groups 1 to 3 (own results).

Groups (n = 8 Specimen Each)	Mean Compression [N]	SD±	Min	Max
1 (<5 mm)	1.75	0.07	1.64	1.86
2 (5.1–7 mm)	0.65	0.03	0.62	0.7
3 (>7.1 mm)	0.26	0.11	0.14	0.45

## Data Availability

Data from the study were unavailable due to privacy restrictions.
